# Low Cost Digital Vibration Meter

**DOI:** 10.6028/jres.112.009

**Published:** 2007-04-01

**Authors:** W. Vance Payne, Jon Geist

**Affiliations:** Building and Fire Research Laboratory National Institute of Standards and Technology, Gaithersburg, MD 20899-8631; National Institute of Standards and Technology, Gaithersburg, MD 20899-8120

**Keywords:** approximate root mean square, cantilever accelerometer, micro electro mechanical system, vibration meter

## Abstract

This report describes the development of a low cost, digital Micro Electro Mechanical System (MEMS) vibration meter that reports an approximation to the RMS acceleration of the vibration to which the vibration meter is subjected. The major mechanical element of this vibration meter is a cantilever beam, which is on the order of 500 µm in length, with a piezoresistor deposited at its base. Vibration of the device in the plane perpendicular to the cantilever beam causes it to bend, which produces a measurable change in the resistance of a piezoresistor. These changes in resistance along with a unique signal-processing scheme are used to determine an approximation to the RMS acceleration sensed by the device.

## 1. Introduction

Vibration monitoring of machinery or components has been recognized as a way to prevent premature or catastrophic failures. The use of vibration monitoring, however, has been historically limited to systems and equipment that are critical to a process or whose failure would result in a considerable loss, because the high cost of industrial grade accelerometers is not an obstacle. Large centrifugal chillers have been using vibration monitoring and analysis to determine when bearings should be replaced or when rotor components were failing. Scaling this type of vibration monitoring technology to smaller residential equipment has not been cost effective.

The use of Micro-Electronic and Mechanical Systems (MEMS) sensors first occurred in the medical, automotive, and process control fields. The automotive industry has truly pushed the use of MEMS sensing technology by using them as air intake gauges, anti-lock braking sensors, and airbag inflation initiators, to name a few [1]. Medical applications for MEMS devices include infusion pumps, dialysis machines, and technology by using them as air intake gauges, anti-lock braking sensors, and airbag inflation initiators, to drug delivery devices [2]. Process control applications use MEMS devices such as accelerometers, gyroscopes, temperature sensors, and pressure sensors. Another widely used MEMS construct has been the ink jet printer head. The ubiquitous nature of MEMS technology will only continue to grow because this technology supplies substantial value through miniaturization, unique performance, improved performance, or reduced cost to every industry that uses it. As the technology matures with the rapid growth of radio frequency MEMS devices and optical MEMS devices, the worldwide market for MEMS technologies is expected to grow from approximately $6.5 billion in 2004 to over $11 billion in 2009 [3].

## 2. Background

### 2.1 MEMS Devices

In general MEMS devices are either sensors or actuators. Examples of the former include pressure transducers and accelerometers, while the later include micro-switches and micro-mirrors. A very good classification and introduction to MEMS technology may be found at the Sandia National Laboratory website [4]. MEMS sensors are further classified by the physics used in their means of transduction; piezoresistive, capacitive, piezoelectric, or vibrational (resonance and acceleration). The National Institute of Standards and Technology (NIST) Building Environment Division vibration meter uses a cantilever beam with a piezoresistor deposited at its base to physically monitor the acceleration of the entire device. The following sections will examine the NIST device and illustrate its operating principles.

### 2.2 The NIST MEMS Vibration Meter

**Fig. 1:**
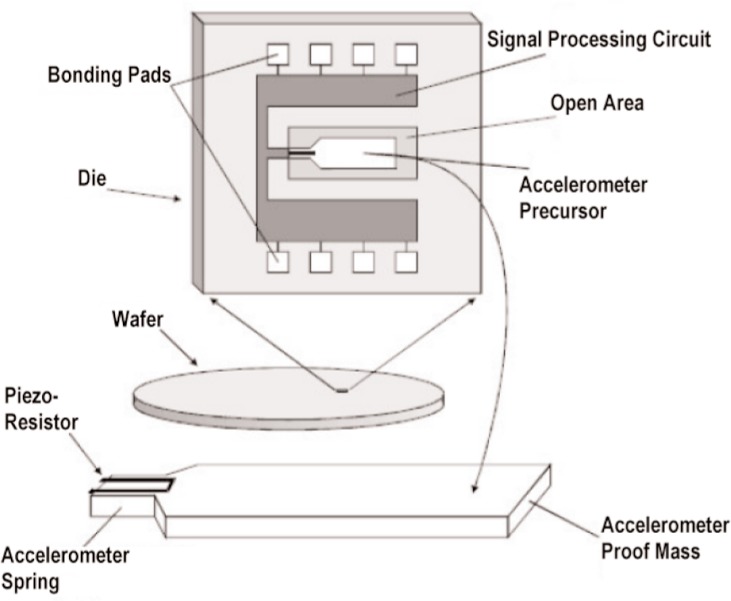
Idealized MEMS die and cantilever beam from a CMOS wafer (The reference cantilever, which is designed to be very insensitive to vibration, but to be sensitive to Joule heating of the cantilever in the same way as the accelerometer cantilever, is not shown for simplicity.)

**Fig. 2:**
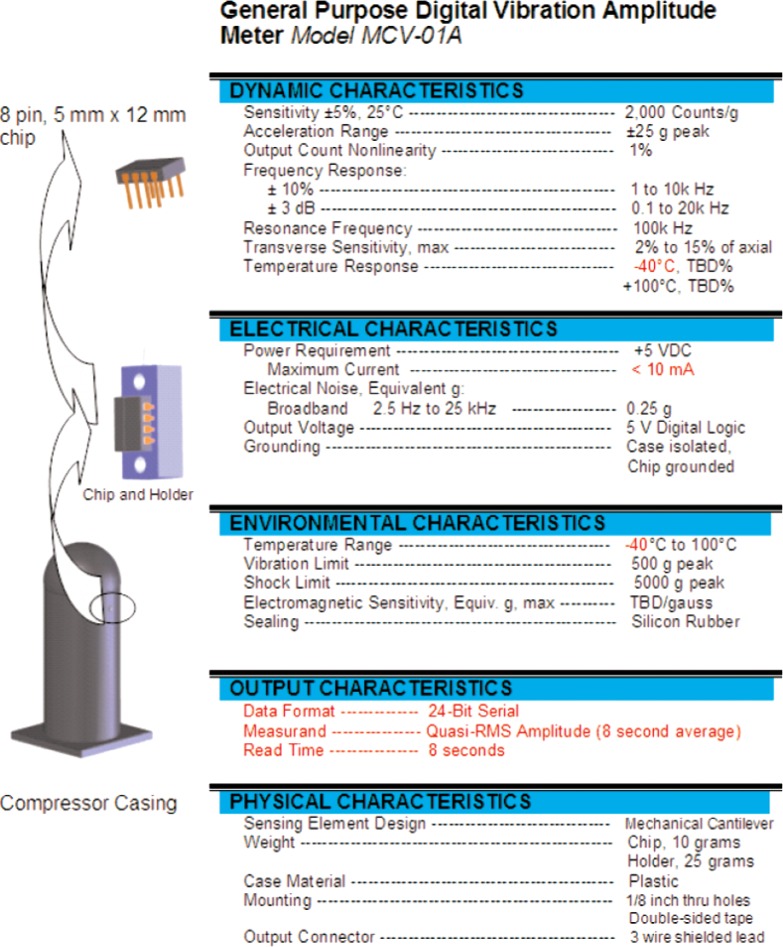
Proposal implementation of the digital vibration meter.

The main goal of NIST’s work with this MEMS device has been to construct a vibration meter that has the necessary sensitivity and accuracy at the lowest possible cost, based upon volumes appropriate to the residential air conditioning market [5]. In order to meet the goals of sensitivity and low cost, standard complimentary metal oxide semiconductor (CMOS) foundry processes were used as the basis for developing a single-chip vibration meter. The CMOS foundry processing techniques utilized for this vibration meter combine the sensor element (cantilever) with the signal processing circuitry on a single chip (Fig. 1). Calibration of the meter is also included in the design and can be performed easily by the end user by flipping the meter upside down with respect to the local gravity field; thereby, producing a change in acceleration from +1 *g* to −1 *g* (a total change of 2 *g*’s). This 2 *g* change in acceleration produces n counts at the meter output. The counts-per-*g* for the meter is then calculated as *n*/2. This operation gives the calibration for a particular chip and allows easy calculation of acceleration. Figure 2 shows a proposed implementation of the vibration meter with a preliminary specification sheet for the device.

## 3. Development of the Vibration Meter Components

**Fig. 3:**
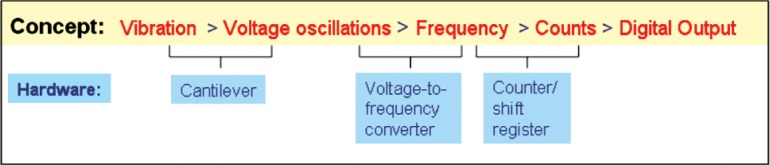
Vibration meter components.

The three main elements of the vibration meter (cantilever, resistance (voltage)-to-frequency converter, and counter/shift register) have been constructed and tested as discrete chips. The main components used to construct the vibration meter are organized as shown in Fig. 3 to convert the acceleration of the cantilever into a discrete digital count stored in a shift register. This count, which is approximately proportional to the root mean square (RMS) value of the oscillatory acceleration in a band centered on a sampling frequency, will be called the ARMS acceleration of the vibration meter. This nomenclature distinguishes it both from an approximation to the instantaneous acceleration that is produced by an accelerometer and from a true RMS measurement of oscillatory acceleration.

### 3.1 Cantilever Accelerometer

**Fig. 4:**
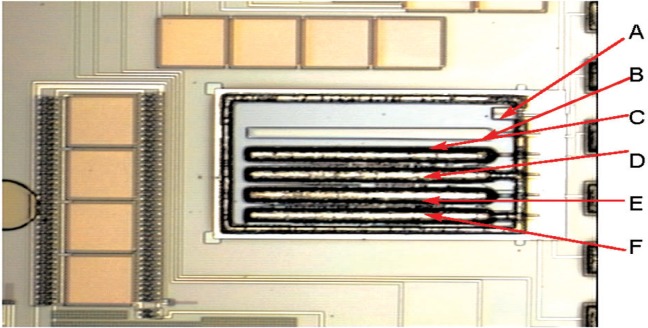
Vibration meter accelerometer cantilever beams.

The mechanical acceleration sensing structure of this device is a cantilever beam. Figure 4 shows a picture of the cantilever-accelerometer beam structures used for development and testing. The figure shows six cantilevers (labeled A to F in Fig. 4) defined by a cut through the CMOS-glass layers that exposes bare silicon before release of the cantilevers by etching in xenon difluoride. Cantilever A was designed to be very insensitive to vibration for use as a reference. Therefore it requires no proof mass and has a wider and therefore stiffer spring. Cantilever B has a proof mass that consists only of the CMOS-glass layers. The next four cantilevers (C to F) have proof masses that consist not only of the CMOS-glass layers but also include a partially exposed CMOS-metal layer over which approximately 6 µm of nickel has been deposited with a commercial electroless-nickel process. Nickel was deposited over the springs of cantilevers D and F to test one potential post process, which did not prove useful. The top-mostCMOS-glass layer was removed from the spring of cantilever-E during chip fabrication to decrease the stiffness of the spring and thereby increase the sensitivity of the cantilever accelerometer.

One terminal of each cantilever’s piezoresistor is connected electrically to a common bonding pad, and the other terminal is connected to a separate bonding pad to allow separate testing of the bottom five cantilevers in a bridge with the reference (top-A) cantilever. Both the addition of the heavier metal to the cantilever proof mass and the reduction in stiffness of the spring increased the vibration sensitivity. Shaker table testing at 30 Hz, 60 Hz and 90 Hz showed that the net increase in peak-to-peak voltage output (sensitivity), compared to the glass-only cantilever with no glass removed from the spring, was about a factor of twenty. The cantilever-accelerometer chips were mounted in a ceramic DIP (Dual Inline Package) with epoxy and the chip pads were wire bonded to the package pads with aluminum wire in a standard IC wedge bonder.

### 3.2 Resistance (Voltage) to Frequency Converter

**Fig. 5:**
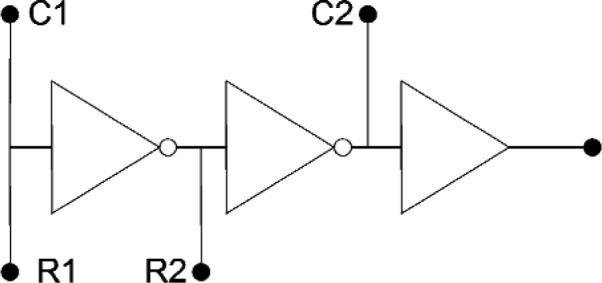
Simplified resistance to frequency converter circuit.

The purpose of the resistance-to-frequency converter is to convert the instantaneous accelerometer piezoresistance to a variable frequency pulse train that can be sampled to estimate the ARMS acceleration. The resistance-to-frequency converter takes the varying resistance value of the vibrating piezoresistor/cantilever and converts it to a 5 V peak-to-peak square wave signal whose frequency is proportional to the connected resistance. To test the fabrication process, a resistance-to-frequency converter was constructed using the same CMOS foundry process used to construct the cantilever accelerometers. A schematic diagram of the resistance-to-frequency converter is shown in Fig. 5. The device was tested by connecting various resistance values across points R1 and R2 and various capacitance values across points C1 and C2 in Fig. 5, and measuring the frequency of the resulting pulse train at the output of the buffer. The results are shown in Table 1. Note that the prototype circuit did not oscillate at all with less than about 6800 Ω connected between points R1 and R2. The results given in Table 1 are consistent with a stray capacitance of *C*_s_ = 20 pF in parallel with the capacitors listed. *C*_s_ was determined by fitting the measured oscillation frequency, *f*, to the following equation.

$$f=K\;R\left(C_{s}+C\right)$$ (1)

where

K is a proportionality constant
R is the resistance connected between points
R1 and R2
C_s_ is the stray capacitance
C is the capacitance connected between points
C1 and C2.

The resistance of the individual cantilever piezoresistors is only about 2000 Ω in the cantilevers shown in Fig. 4. Therefore, at least three or more piezoresistors, which are mechanically in parallel when located on the same IC die, would have to be connected in electrical series to provide the required resistance for the resistance-to-frequency converter. Therefore, it may prove more practical to configure the reference and sensor accelerometers in a bridge, to amplify the AC of the bridge output, and use a conventional voltage-to-frequency converter to produce the variable frequency pulse train.

### 3.3 Counter/Shift Register Chip

**Fig. 6:**
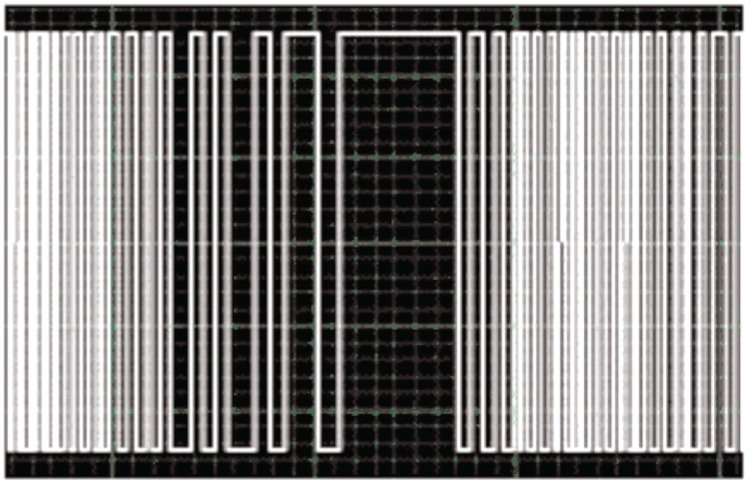
Counter/shift register chip input.

A simple 4-channel counter chip with an onboard output shift register was also designed and fabricated using the same CMOS techniques used to fabricate the cantilever accelerometers and resistance-to-frequency chips. Figure 6 shows a typical pulse train being sent from the resistance-to-frequency converter output to the counter’s parallel tied inputs. Transitions of the pulse trained were registered as a positive increment of the counter. The main goal of this test chip was to determine the functionality versus size trade-offs in producing the required signal processing circuits on the same chip as the accelerometer and to verify that the circuits produced from the available design libraries functioned as expected.

## 4. Theory of Operation

**Fig. 7:**
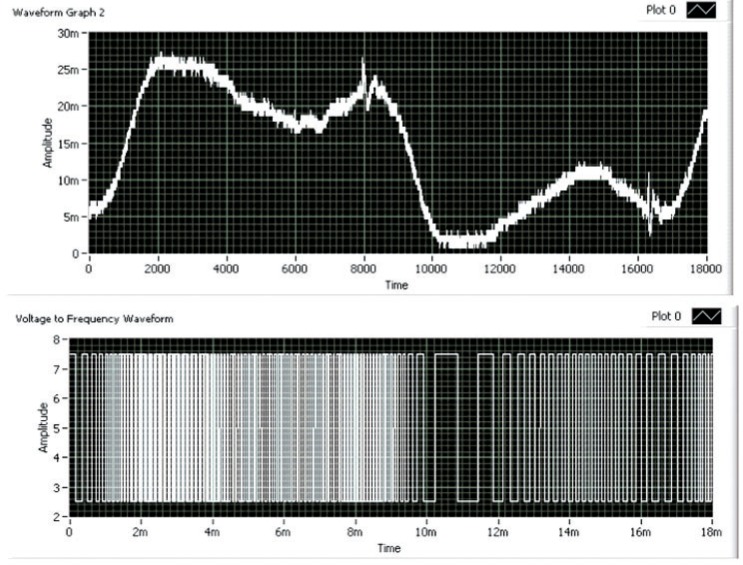
Cantilever accelerometer voltage signal converted to variable frequency pulse train.

The cantilever accelerometer chip was initially tested using a software emulation of the ARMS acceleration meter's logic chips. The piezoresistive accelerometer was connected to the reference accelerometer in a bridge and AC coupled to a commercial operational amplifier connected in the voltage amplification mode. A data acquisition program was written to convert the varying analog voltage signal coming from the vibrating cantilever chip to a varying frequency square wave or pulse train as shown in Fig. 7. The square wave frequency was equal to a constant (10e+6) multiplied by the digitized voltage. The voltage offset was subtracted from the signal in the software to yield a waveform close to the origin. In reality the voltage offset would add to the waveform voltage signal sent to the voltage-to-frequency converter increasing the voltage magnitude but having no effect on the number of counts at the output. The analog voltage signal was measured using a PCI card digital oscilloscope. This conversion is the software equivalent to the resistance-to-frequency chip described above. Both the operational amplifier and the voltage to frequency converter can be easily integrated on the same CMOS chip with the accelerometer, but the AC coupling capacitor was far too large for on-chip integration. Either a separate capacitor chip (incorporated within the chip holder of Fig. 2) or a much more complicated on-chip differential amplifier would be required. These are the dis-advantages of this approach compared to using a three-cantilever accelerometer with the CMOS resistance-to-frequency converter described above.

The peak-to-peak amplitude of the voltage wave shown in Fig. 7 is proportional to the ARMS acceleration of the chip. Also note in Fig. 7 that locations of peak acceleration produce areas of higher frequency within the associated pulse train. The acceleration zero point is near the center of the voltage trace, and the point of peak negative acceleration produces the lowest voltage and thus lowest frequency within the associated square wave.

Given a pulse train similar to that shown in Fig. 7, the next step in determining the ARMS acceleration is the implementation of a suitable pulse-counting scheme. The key concept constraint that allows this voltage to frequency counting scheme to measure approximate RMS acceleration is the knowledge of the vibration frequency at which you wish to determine the associated ARMS acceleration. This allows simple (and cheap to construct, i.e., small die area) flip/flop counters to be used in measuring ARMS acceleration. The main emphasis with this technique is the use of flip/flop counters, which results in a low-complexity vibration-amplitude measurement device.

With the frequency of the ARMS acceleration known, the technique seeks to sample this vibration waveform for a length of time approximately equal to or slightly greater than the vibration’s period (the reciprocal of the frequency). For a given oscillatory acceleration, this sampling technique will produce the maximum count in a broad frequency range in the vicinity of the sampling frequency and will produce lesser counts at other vibration frequencies. The sampling technique utilized is calibrated at the frequency of the vibration at which the corresponding amplitude is measured, and it will ignore other vibration frequencies unless they are harmonics of the indicated frequency of interest.

**Fig. 8:**
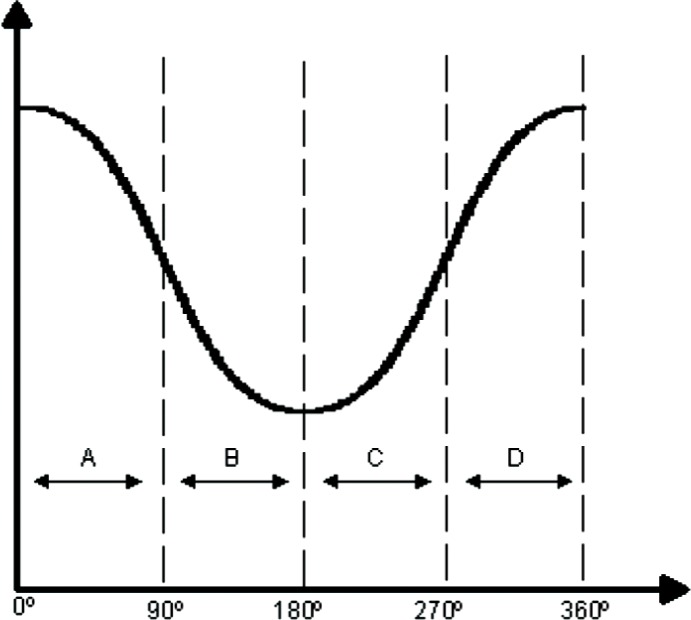
Example sine wave broken into four regions.

For example, let us examine a sinusoidal vibration shown in Fig. 8 occurring at 60 Hz. The counting scheme takes a snapshot of the associated variable frequency pulse train for a length of time equal to the period of the vibration frequency of interest. For this example this sample interval would be 16.67 ms. The scheme then implements a differencing technique to calculate the total number of “counts” associated with this particular waveform. If we look at the sinusoidal wave in Fig. 8, the differencing scheme seeks to take absolute differences of the various counts occurring at 90° intervals according to the following formula.

$$\begin{array}{l} Total\;Counts=\\ \left|Counts\left(A\right)-Counts\left(C\right)\right|+\left|Counts\left(B\right)-Counts\left(D\right)\right| \end{array}$$ (2)

where

*Counts (A)* = total counts occurring in the time interval from 0° to 90°
*Counts (B)* = total counts occurring in the time interval from 90° to 180°
*Counts (C)* = total counts occurring in the time interval from 180° to 270°
*Counts (D)* = total counts occurring in the time interval from 270° to 360°

**Fig. 9:**
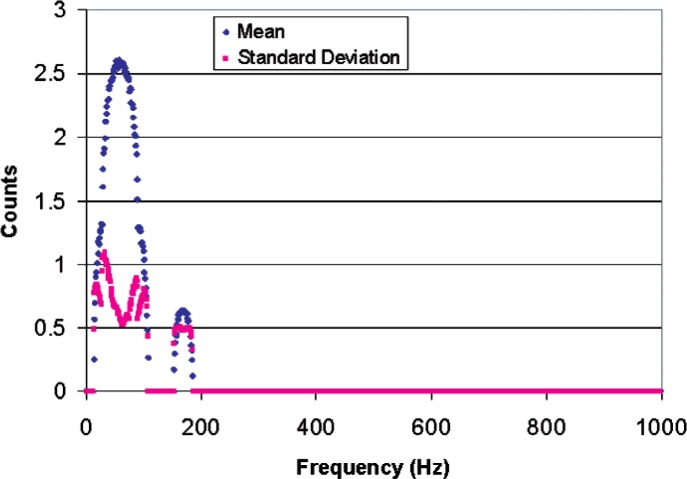
Simulated sampling scheme for a 60 Hz, 400 counts/ms amplitude vibration mixed with 1000 samples of random phase at a 10 MHz counting rate.

The counting logic breaks the vibration’s pulse train into four equal time interval pulse trains corresponding to regions A, B, C, and D of Figure 8. These pulse trains are “counted” by four different counters and the resulting “Total Counts” is determined according to Eq. (2). The sampling scheme of Eq. (2) and Fig. 8 was simulated for a vibration frequency of 1 Hz to 1000 Hz with 1000 samples of random phase at each frequency. The length of the individual sample periods A, B, C, and D were 4 ms long to yield a vibration period of 16 ms (62.5 Hz ~ 60 Hz). The average frequency of the frequency-modulated pulse train that is counted during each sample period was assumed to be 10 MHz. This frequency will provide a count of 40,000 in each of sample periods A, B, C, and D in the absence of vibration (zero-amplitude vibration), which will result in zero counts from the sampling scheme of Eq. 2. Three simulations were run with modulation amplitudes of 1 %, 10 %, and 100 %, which produce plus or minus 400 counts, plus or minus 4,000 counts, and plus or minus 40,000 counts, respectively. Figure 9 shows the average number of counts and standard deviation over the 16 ms sample interval produced by the counting scheme for vibrations ranging in frequency from 1 Hz to 1000 Hz with a vibration amplitude that produced a modulation amplitude of 1 %. Notice that the standard deviation of a single sample is a substantial fraction of the sample average, but there will be about 62.5 samples per second, which reduces the standard deviation by a factor of 7.9 for one-second averages, and by a factor of 61 for one-minute averages.

**Fig. 10:**
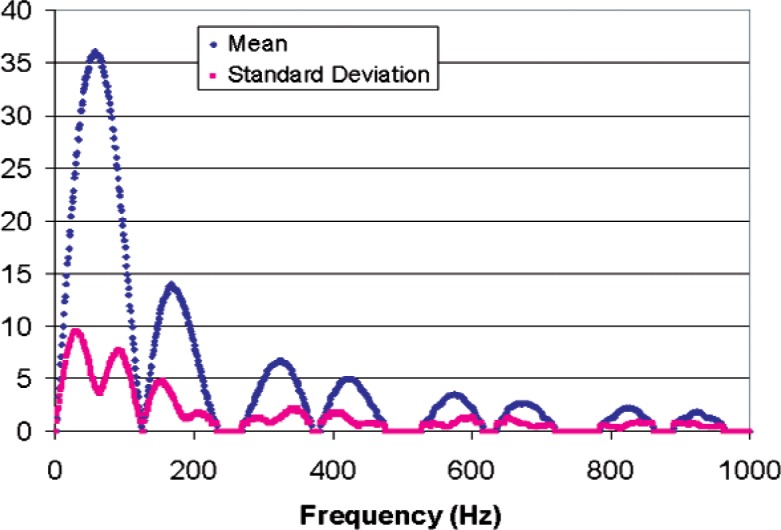
Simulated sampling scheme for a 62.5 Hz, 4000 counts/ms amplitude vibration mixed with 1000 samples of random phase at a 10 MHz counting rate.

**Fig. 11:**
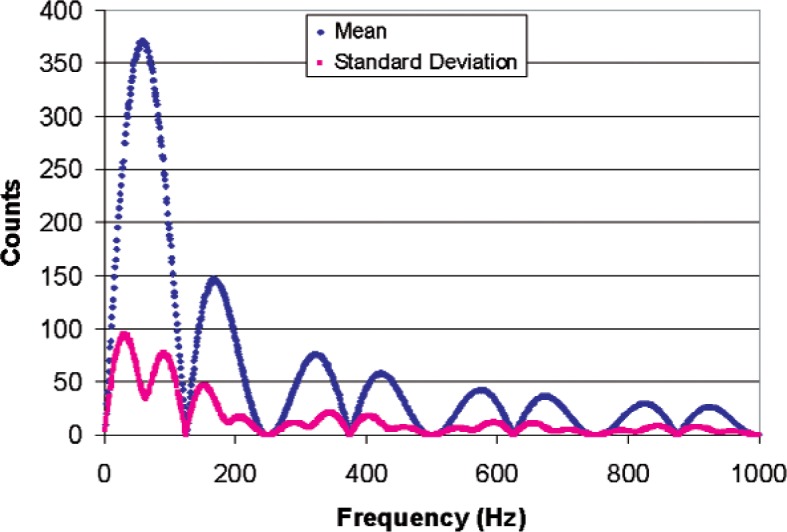
Simulated sampling scheme for a 62.5 Hz, 40000 counts/ms amplitude vibration mixed with 1000 samples of random phase at a 10 MHz counting rate.

Figure 10 shows the average number of counts and standard deviation over the 16 ms sample interval produced by the counting scheme with a modulation amplitude of 10 %. Similarly, Fig. 11 shows the same results for a modulation amplitude of 100 %. If the modulation amplitude is reduced below about 0.05 %, the number of counts falls to zero, and modulation amplitudes of more than 100 % are not physically realizable. Therefore the practical dynamic range of this counting scheme in a small band centered on the vibration frequency is of the order of 100. Comparison of Figs. 9 and 11 shows that it is even smaller for sampling frequencies further removed from the vibration frequencies. However, there are applications for which a small dynamic range is sufficient, such as detecting vibration above a threshold level.

## 5. Example Test Results

Testing of the various cantilever accelerometers was performed using a shaker table setup calibrated at four different vibration frequencies over a range of peak acceleration. Table 2 below lists the calibration frequencies, total acceleration range, and calibration accelerometer specifications. A waveform generator was connected to the shaker table amplifier and supplied a sine wave signal at the listed frequencies. The voltage signal from the waveform generator was monitored by a PCI computer bus digital oscilloscope card and characterized by peak-to-peak voltage and RMS voltage.

**Fig. 12:**
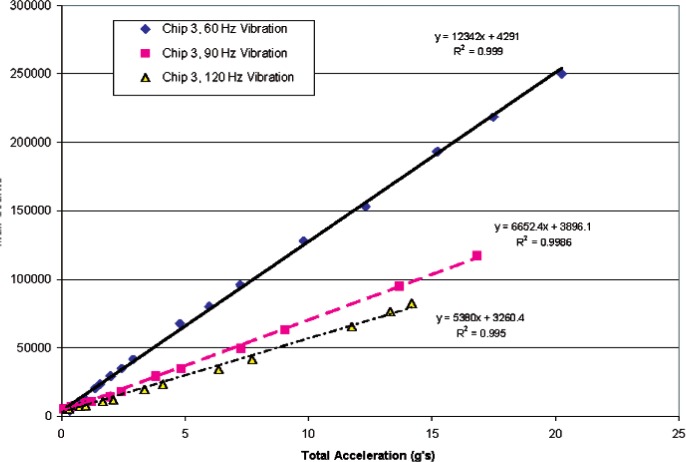
Cantilever accelerometer tests with software counting scheme at three vibration frequencies.

**Fig. 13:**
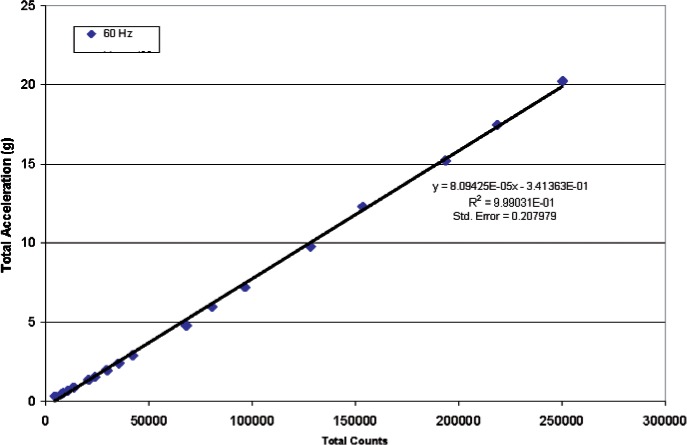
ARMS acceleration as a function of total counts at a 60 Hz vibration frequency.

Figure 12 shows the results of testing the most sensitive metal-coated accelerometer of the same design as seen in Fig. 4 with the software counting scheme. The ARMS acceleration was shown to be linear with respect to the total counts determined from Eq. (1). If this particular metal-coated cantilever accelerometer were used in a ARMS acceleration meter, the associated acceleration amplitude as a function of the number of counts is shown in Fig. 13. The standard error of the linear fit in Fig. 13 shows that this accelerometer combined with the software counting scheme could produce uncertainties of ± 0.42 *g* at a confidence level of 95 %. This is in addition to the calibration uncertainty of approximately ± 0.5 *g* from shaker table standard error as shown in Table 2.

## 6. Wafer Post Processing and Die Packaging the Vibration Meter as a Single Chip

### 6.1 Concept

All three of the functional sub-sections of the components of the vibration meter are constructed on a single common die using standard CMOS techniques. The mechanical cantilevers and all electrical components/circuitry are constructed on standard size silicon wafers, post processed to deposit nickel on the exposed aluminum on the cantilever, mounted in a DIP package, wire bonded, and further post processed to release the cantilevers to vibrate in response to vibration of the package.

### 6.2 Wafer Post-Processing

The production of a low cost vibration meter requires the use of low-cost post-processing and low cost packaging, as well as standard CMOS fabrication technology. The nickel electroless deposition process used for the devices described here can be carried out as a very low-cost process at the wafer level. It requires dipping the wafers in a room temperature, low-cost commercial solution of a zinc-salt for 5 s to 15 s and in a 95 °C, low-cost commercial nickel-salt solution for 5 min to 15 min. Because the cantilevers are not released from the silicon-die substrate at this point, the dies sawed from the wafers can be packaged with any standard IC packaging process. Finally, the entire package can be exposed to xenon di-fluoride or bromine pentafluoride to release the cantilevers by creating an etch pit under them.

### 6.3 Packaging

**Fig. 14:**
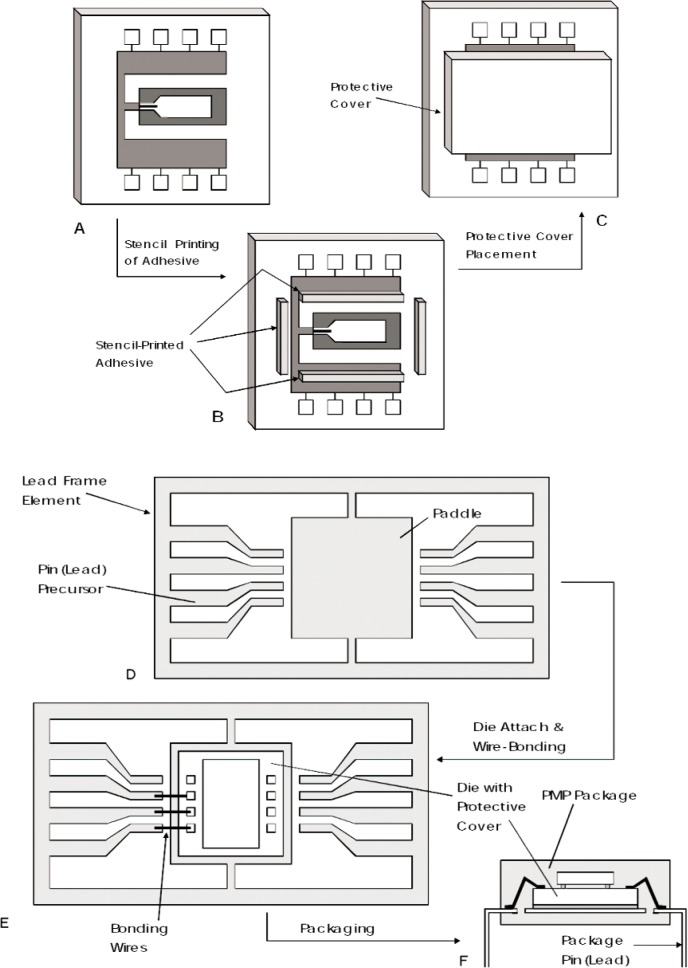
Illustration of a packaged vibration meter in single chip form.

Unfortunately, IC packaging is quite expensive except in very large volumes. Currently, Post Molded Plastic (POMP or PMP) packaging is the lowest cost packaging technique for low pin count chips with an average cost of 1 cent per pin at a volume of 10 000 chips per week. In order to take advantage of this low cost packaging process, an economical vibration meter cover installation technique must be developed to produce a packaged vibration meter on a single chip as shown in Fig. 14. The vibration meter needs this cover to allow for free vibration and to protect the cantilevers from outside damage.

**Fig. 15:**
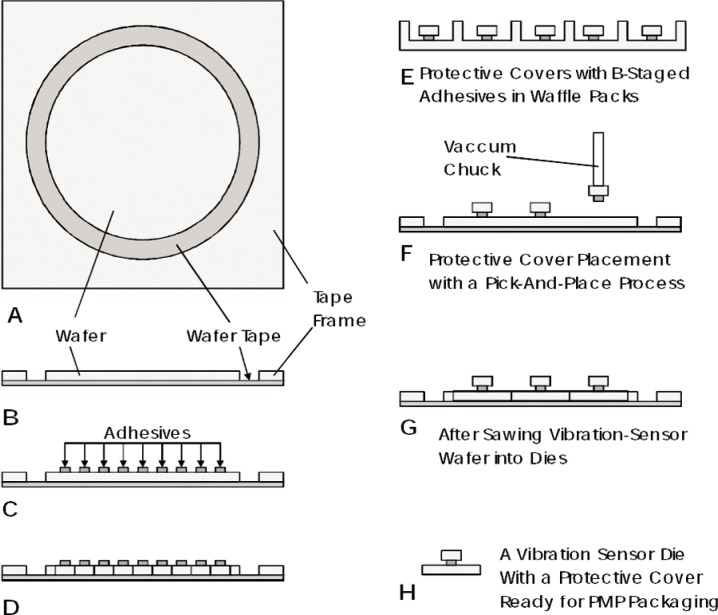
Pick-and-place vibration meter cover placement technique.

NIST has studied methods to apply covers to the vibration meter dies using commonly available processing methods [6]. One such sequence involved using industry standard B-stage processing techniques enumerated below and illustrated in Fig. 15:

1. Adhesive rings are stenciled onto the protective covers before the covers are sawed into individual dies (Fig. 15C).
2. The wafer containing all the covers is heated only enough to remove the tackiness from the cover adhesive.
3. The cooled wafer containing the adhesive stenciled covers is sawed to produce individual adhesive cover dies (Fig. 15D).
4. The protective covers are then picked from the wafer tape and placed on the vibration meter wafer while the vibration meter is heated sufficiently to make the adhesive tacky (Fig. 15F).
5. The vibration meter wafer is then heated to completely cure the adhesive rings and secure the protective covers (Fig. 15G).

The adhesive used to secure the protective covers was investigated, and a suitable product was found. A completely automated process for performing the cover placement was outlined by Geist [6], but he also suggested examining a different packaging technique.

PREmolded Plastic packaging (PREMP) was also examined as a packaging technique, but its cost is approximately twice that of the PMP packaging examined above (2 cents per pin). The higher cost of this packaging process, however, may be offset by the lowered cost of adding protective covers. The PREMP packaging process produces an open chip with no protective cover over the centrally located vibration meter die. The vibration meter dies are packaged in this open PREMP package before any nickel coating or etching is performed. The nickel coating is performed, then the cantilevers are released to vibrate freely while the entire die is in the PREMP package. Protective covers are made separately and sawed to fit the PREMP package. These covers are then attached by hand. The main reason that the PREMP packaging may be less expensive than the PMP packaging is that the protective covers for the PREMP packaging require less precise alignment, and handling a packaged die is much easier than handling the individual dies alone. Also, since precision placement of the protective covers is not required, less expensive pick-and-place machines may be used to automate the cover placement process.

Geist [6] concluded that the PMP packaging technique would cost the least, especially at chip volumes above 500 000 units. At volumes of 5 million chips per year, the expected chip cost was estimated at under $2 per chip. The PREMP packaging technique was not examined directly for the vibration meter, but this packaging technique was used by the author for nominally the same sized MEMS devices (2 mm × 2 mm). The PREMP packaging technique, which is suggested in this report, is currently less expensive than PMP packaging at volumes less than 2 million chips. Furthermore, the price is likely to decrease significantly. Many MEMS devices, such as gas sensors, will not function if hermetically sealed. Therefore, the demand for lower cost open packages will grow as various types of MEMS devices become more widely deployed. PREMP packaging is ideally suited to meet this demand and should ultimately cost much less at any given volume because a hermetic seal is not required. At the same time many new passivation techniques such as nitride or parylene deposition have been, and are being, developed to allow ICs to perform satisfactorily in non-sealed environments.

## 7. Summary

The ARMS acceleration meter outlined in this report is by no means the only method of determining the RMS acceleration of vibrations. It is the intent of this technique to produce a low cost device that would be economically suitable for installation in residential size heat pumps and air conditioning equipment. This type of device would be used to monitor compressor, fan, or rotating equipment performance within the system [7]. The digital output of this type of device could be easily interfaced with most microprocessors used today in air conditioning controls. The economics of manufacturing this meter are yet to be proved, but the investigators believe that simple components and CMOS foundry techniques will produce the lowest cost meter possible.

Most compressor and component manufacturers know how their devices fail and have measured the associated vibration waveforms during failures. Using the knowledge of known “problem” frequencies, the NIST vibration meter could monitor those vibrations and indicate a problem before system failure occurred. It is the intention of this development effort to produce a single-chip vibration meter and test it on real compressors and rotating equipment used in air conditioning equipment. The final chip design, CMOS foundry processes, and packaging techniques will determine the economic viability of this single-chip meter. A full range of shaker table testing will determine the characteristics of the single-chip meter.

## Figures and Tables

**Table 1:** Resistance-to-frequency converter characteristics

R (Ω)	C (F)	f (Hz)
6830	1.50 E−10	3.40 E + 05
6830	5.60 E−11	7.46 E + 05
6830	1.20 E−11	1.70 E + 06
6830	5.00 E−12	2.23 E + 06
7400	5.00 E−12	2.06 E + 06
8950	5.00 E−12	1.82 E + 06

**Table 2:** Calibration test performance parameters

Calibration Frequency (Hz)	Maximum Total Acceleration (g)	Total Acceleration Linear Fit Standard Error (g)^a^
60	26	0.27
90	24	0.32
120	20	0.39

mV/g	Quartz Accelerometer Specifications Range	Offset Voltage (V)

101.3	± 50 g	8.30

a Linear fit equation: Total Acceleration (g) = slope × Shaker Input Voltage (mV) + intercept.

## Nomenclature

AC: alternating current
DIP: dual inline package
*g*: acceleration in earth’s gravity field (9.8 m/s^2^)
IC: integrated circuit
RMS: root mean squared
ARMS: approximate RMS acceleration in a frequency band
